# Predictors of the extended-spectrum-beta lactamases producing Enterobacteriaceae neonatal sepsis at a tertiary hospital, Tanzania

**DOI:** 10.1016/j.ijmm.2018.06.012

**Published:** 2018-10

**Authors:** Rehema Marando, Jeremiah Seni, Mariam M. Mirambo, Linda Falgenhauer, Nyambura Moremi, Martha F. Mushi, Neema Kayange, Festo Manyama, Can Imirzalioglu, Trinad Chakraborty, Stephen E. Mshana

**Affiliations:** a Department of Pediatrics and Child Health, Weill Bugando School of Medicine, P.O. Box 1464, Mwanza, Tanzania; b Department of Microbiology and Immunology, Weill Bugando School of Medicine, P.O. Box 1464, Mwanza, Tanzania; c Institute of Medical Microbiology, Justus-Liebig University, Schubertstrasse 81, 35392, Giessen, Germany; d German Center for Infection Research (DZIF), Partner site Giessen-Marburg-Langen, Campus Giessen, Schubertstrasse 81, 35392, Giessen, Germany

**Keywords:** Neonatal sepsis, ESBL-PE, Predictors, *Klebsiella pneumoniae*, ST45

## Abstract

The study was conducted to establish predictors of extended-spectrum beta-lactamase-producing Enterobacteriaceae (ESBL-PE) neonatal sepsis and mortality in a tertiary hospital, Tanzania. Between July and December 2016, blood culture was performed in neonates with clinical features of sepsis and neonates/mothers/guardians were screened for ESBL colonization. Selected isolates underwent whole genome sequencing to investigate relatedness. Logistic regression analysis was performed to determine predictors for ESBL-PE associated neonatal sepsis and mortality. Neonatal ESBL-PE sepsis was detected in 32(10.5%) of the 304 neonates investigated. Neonatal ESBL-PE sepsis was independently predicted by admission at the Intensive care Unit and positive mother and neonate ESBL-PE colonization. Deaths occurred in 55(18.1%) of neonates. Neonates infected with ESBL-PE, admitted at ICU, increased age and those transferred from other centres had significantly high mortality rates. Gram-negative bacteria formed the majority (76%) of the isolates, of which 77% were ESBL-PE. Virulent *Klebsiella pneumoniae* ST45 carrying *bla*_CTX-M-15_ were commonly isolated from neonates. *Klebsiella pneumoniae* (ST45) were the predominant cause of ESBL-PE neonatal sepsis and mortality. Improved infection control and antibiotic stewardship are crucial in controlling the spread of resistant strains. Rapid diagnostic tests to detect ESBL-PE in low-income countries are needed to guide treatment and reduce ESBL-PE-associated mortality.

## Highlights

• *ESBL-PE sepsis was predicted by admission at ICU* and ESBL-PE colonization.
• Neonates infected with ESBL-PE had significantly high mortality.
• ESBL-producing *Klebsiella pneumoniae* (ST45) carrying *bla*_CTX-M-15_ were predominant.
• Whole genome SNP analysis revealed clonal origin in 50% of ESBL-PE paired cases with similar sequence type.

## 1 Introduction

Neonatal sepsis is one of the top three causes of morbidity and mortality during the neonatal period (Bhutta et al., 2010). Diagnosis and management of sepsis pose a great challenge for neonatologists in NICUs especially in low-income countries (Sharma et al., 2016). The increase of multidrug-resistant organisms in neonatal units limits the treatment options and delays effective treatment, resulting in increased morbidity and mortality (Patel and Saiman, 2010). In many low-income countries including Tanzania, extended-spectrum beta-lactamase-producing Enterobacteriaceae (ESBL-PE) are the commonest cause of neonatal sepsis (Christopher et al., 2013; D’Andrea et al., 2013; Ghotaslou et al., 2007; Mshana et al., 2009; Thaver et al., 2009). Infections due to ESBL-PE are associated with increased morbidity and mortality (Blomberg et al., 2005; Kayange et al., 2010; Mhada et al., 2012). The antibiotics of choice for the treatment of neonatal sepsis due to ESBL-PE pathogens are not available in most settings and even when stocked, they are too expensive to be afforded (Vandijck et al., 2008). Varieties of ESBL-PE genotypes have been found in humans, animals and the environment in the city of Mwanza, Tanzania (Mshana et al., 2016). Despite ESBL-PE being prevalent in the city of Mwanza, the transmission pathways and risk factors of these pathogens in relation to neonatal sepsis is not well studied. This study was undertaken to investigate factors associated with ESBL-PE neonatal sepsis and mortality among neonates admitted at the Bugando Medical Centre (BMC), Mwanza, Tanzania. In addition, the study aimed to characterize selected isolates to show their virulence potential and transmission dynamics.

## 2 Material and methods

### 2.1 Study duration, area and inclusion criteria

The study was conducted between July and December 2016 at the premature unit and neonatal Intensive care unit (NICU) of BMC. All neonates (0–28 days) with signs and symptoms of sepsis were enrolled. The sample size was estimated using the Kish Leslie formula for the cross sectional studies (Kish, 1965).

### 2.2 Data collection

Using signs and symptoms published by “The WHO Young infants Study group” (Margolis et al., 1999), a data collection tool was designed and used to obtain socio-demographic and clinical data together with other relevant factors related to risks for neonatal sepsis. At the time of enrollment, clinical data and blood samples were collected. Antenatal history such as use of antibiotics, presence of chronic diseases, febrile illness during pregnancy, labor history, duration of labor, history of premature rupture of membrane, mode of delivery were recorded. Neonates were subjected to a full clinical examination to assess temperature, respiration rate, presence or absence of cyanosis, jaundice, umbilical redness, convulsion, reduced movement and ability to feed.

### 2.3 Microbiological procedures

Umbilical-rectal swabs were collected using sterile cotton swabs in Amies transport medium (Biolab, HUNGARY) from all neonates to determine colonization status. Analysis of stool samples obtained from mothers/guardians provided data on colonization status. Swab and stool samples were inoculated on MacConkey agar (Oxoid, UK) supplemented with 2 μg/ml cefotaxime (Medochemie Ltd, Cyprus EU) and processed as previously described (Nelson et al., 2014). Using aseptic techniques approximately 1.5–2 ml of blood was obtained and inoculated directly into Brain Heart Infusion broth (BHI) (Oxoid Ltd, UK) in a ratio of blood to BHI of 1:10 and transported to the Catholic University of Health and allied Sciences (CUHAS) Microbiology laboratory for incubation and subsequent processing as previously described (Kayange et al., 2010). All blood culture isolates were tested for their antibiotic susceptibility pattern using disc diffusion methods and interpreted following the Clinical Laboratory Standard Institute guidelines (CLSI, 2015). Confirmation of the ESBL phenotype was performed using the disk approximation method (Mshana et al., 2009). All neonates enrolled in the study were assessed and managed adhering to the BMC neonatal unit protocols. The first line antibiotic treatment option included ampicillin and gentamicin, the second line included cefotaxime followed by the third line that incorporated the use of meropenem. All neonates were evaluated on a daily basis prior to discharge, after which they were monitored for a further 28 days.

### 2.4 Whole genome sequencing and *in silico* analyses

Fifty-three (16 from blood and 37 from colonized neonates) ESBL-PE isolates were subjected for whole genome sequencing (WGS). These isolates were serially selected as they were isolated, no other criteria were used, and aim was to sequence the first 50 isolates. DNA was isolated using PureLink® Pro 96 Genomic DNA Purification Kit (Thermo-Fisher Scientific, Dreieich, Germany) according to the manufacturer's instruction. WGS was performed on an Illumina NextSeq 500 instrument (Illumina, San Diego, CA, USA) using an Illumina Nextera XT library with 2 × 150bp paired-end reads. The data was assembled using SPAdes (version 3.6.2) (Bankevich et al., 2012).

Sequences were analyzed for their multi locus sequence types, transferrable resistance genes, plasmid replicon types and pMLST using MLST 1.8, ResFinder, Plasmidfinder and pMLST software of the Center for Genomic Epidemiology (https://cge.cbs.dtu.dk/services/), respectively (Carattoli et al., 2014; Larsen et al., 2012; Zankari et al., 2012). The presence/absence of *Klebsiella pneumoniae* virulence genes was assessed using blastn following Holt et al (Holt et al., 2015).

The SNP analysis of isolates depicting identical STs was performed using SAMtools (Li, 2011). Regions of high recombination (>50 SNPs per 1 kb) were excluded manually from the analysis.

The raw data of all sequenced ESBL-PE isolates are available at the European Nucleotide Archive (ENA) under the project number PRJEB20875.

### 2.5 Ethics approval

This study obtained clearance from the Joint CUHAS-BMC Ethics and Scientific Review committee with certificate no: CREC/162/2016. Written informed consent was obtained from parents/guardians of the study participants.

### 2.6 Statistical analysis

Data was entered using Microsoft Excel 2007 and processed using STATA version 13.0 (Stata Corp, College Station, TX, USA). Categorical variables such as history of antibiotic use, history of admission, colonization status etc., were presented proportionally and compared using chi squared test or Fisher’s exact test. Continuous variables such age, gestation age, length of hospital stay, weight etc., were described as medians (inter quartile range/ IQR)). To determine predictors of ESBL-PE neonatal sepsis, ESBL-PE neonatal colonization and the outcome, univariate followed by multivariate logistic regression analysis was performed. All factors with p value of less than 0.2 were further subjected to multivariate logistic analysis and controlled for both age and sex. To estimate survival rates and hazard ratios, a Cox regression model was used. All factors with p value less than 0.05 at 95% confidence interval (CI) were considered statistically significant.

## 3 Results

### 3.1 Demographic and clinical characteristics

A total of 304 neonates were enrolled in the study. Male neonates formed the majority 192 (63.2%) of study participants. The median age of the enrolled neonates was 6 days (IQR: 3–9). A total of 94 (30.1%) neonates had birth weights of below 2.5 kg. The median gestation age was 39 weeks (IQR: 37–40) and the median body temperature measured was 37.9 °C (IQR-37-38.2 °C). A total of 57 (14.1%) of all neonates were delivered by cesarean section (C/S) and 16 (5.3%) were delivered at home (Table 1).

**Table 1:** Socio-demographic and clinical characteristics of 304 neonates with clinical sepsis.

Neonate Characteristics	Number	Percent (%)/Median
**Age (days)**	304	6 (IQR: 3-9)
**Body temperature**	304	37.85 (IQR:37-38.2)
***Oxygen Saturation %***	304	93 (IQR: 90-96)
***Birth weight(Kg)***	304	2.9 (IQR: 2.2-3.2)
***Admissions***		
*BMC*	116	38.2
*Referral*	188	61.8
***Sex***		
Female	112	36.8
Male	192	63.2
***Mode of Delivery***		
Cesarean section	57	14.1
Vaginal delivery	247	85.9
***Age*** (days)		
≤7	111	36.5
>7	193	63.5
***Birth Weight***		
< 2.5 kg	94	30.1
>2.5 kg	210	69.1
***Gestation Age***		
Full Term	233	76.6
Premature	71	23.4
***Body temperature***		
< 37.5 °C	120	39.5
≥37.5 °C	184	60.5
***Convulsion***		
No	263	86.5
Yes	41	13.5
***Sucking***		
No	164	53.9
Yes	140	46.1
***Skin pustule***		
No	257	84.5
Yes	47	15.5
***Lethargy***		
No	197	64.8
Yes	107	35.2
***Cyanosis***		
No	251	82.6
Yes	53	17.4
***Jaundice***		
No	220	72.4
Yes	84	27.6
***Umbilical discharge***		
No	243	79.9
Yes	61	20.1

### 3.2 ESBL-PE neonatal sepsis, colonization, isolates and genotypes

Of 304 neonates, 32 (10.5%, 95%CI: 7.1–13.9) were infected by ESBL-PE. Neonatal ESBL-PE colonization occurred in 166 cases (54.6%; 95%CI: 49–60.1). In total, 86 (28.3%, 95%CI: 23–33.3) of mothers/guardians were carriers of ESBL-PE. Of the 32 neonates infected by ESBL-PE, *Klebsiella pneumoniae* was the most common pathogen (65.6%) (Table 2). This was also the case for neonate colonization, where *Klebsiella pneumoniae* isolates was the most common ESBL-PE (n = 112, 67.5%). On the other hand, of 86 mothers/guardians colonized by ESBL-PE, only 26 (30.2%) were colonized by ESBL-producing *Klebsiella pneumoniae*. Neonates were significantly more often infected/colonized by *Klebsiella pneumoniae* than guardians/mothers (P < 0.001). Other bacterial species isolated from the blood of the neonates were *Enterococcus* spp., *Staphylococcus aureus*, *Streptococcus pyogenes*, *Escherichia coli*, *Acinetobacter baumannii*, *Salmonella* spp., *Citrobacter* spp., and *Enterobacter* spp. (Table 2). The proportion of the *Klebsiella pneumoniae* isolates (n = 26) resistant to ampicillin, amoxicillin/clavulanic, gentamicin, cefotaxime, ciprofloxacin, amikacin and meropenem was 100%, 80%, 84%, 80%, 40%, 60% and 0%, respectively. For the other Gram-negative bacteria (n = 19), the proportion of isolates resistant to ampicillin, gentamicin, cefotaxime, ciprofloxacin, amikacin, amoxicillin/clavulanic and meropenem was 94%, 80%, 79%, 56%, 28%, 70% and 10.5%, respectively. The two isolates that were resistant to meropenem were *Acinetobacter baumanii*. All of the seven *Staphylococcus aureus* were methicillin-sensitive, and all three *Streptococcus pyogenes* isolates were sensitive to penicillin.

**Table 2:** Distribution of ESBL-producing isolates from blood, colonizing neonates and those colonizing mothers.

Isolate	Blood (N = 60)	ESBL from Blood(N = 32)	ESBL Colonizing neonates(N = 178)	ESBL colonizing mothers(N = 86)
*Klebsiella pneumoniae*	26 (43%)	21 (65.6%)	112 (67.5%)	26 (30.2%)
*Escherichia coli*	3 (5.0%)	–	60 (36.1%)	60 (69.8%)
*Acinetobacter baumannii*	9 (16%)	7 (21.9%)	5 (3.0%)	–
*Citrobacter* spp.	2 (3.3%)	2 (6.3%)	–	–
*Enterobacter* spp.	2 (3.3%)	2 (6.3%)	1 (0.6%)	–
*Pseudomonas aeruginosa*	2 (3.3%)	NA	NA	NA
*Salmonella* spp.	1 (1.6%)	NA	NA	NA
*Enterococcus* spp.	5 (8.3%)	NA	NA	NA
*Staphylococcus aureus*	7 (11.6%)	NA	NA	NA
*Streptococcus pyogenes*	3 (5%)	NA	NA	NA

NA = Not applicable.

### 3.3 Data from whole genome sequencing analysis

Out 296 phenotypically confirmed ESBL isolates, 53(17.9%) were subjected to whole genome sequencing, 38 (68%) were *Klebsiella pneumonia* of which 15 originated from blood cultures and 23 from neonatal swabs. *Klebsiella pneumoniae* with the sequence type (ST) ST45 and carrying the *bla*_CTX-M-15_ was predominant in blood- (7/15) and from swab- cultures (11/23). Other *Klebsiella pneumoniae* STs observed were ST348 (3), ST973, ST17, ST20, ST711, ST101, ST14, ST2268 and ST35 (Table 3). All *Klebsiella pneumoniae* isolates carried the *bla*_CTX-M-15_ allele. Quinolone resistance genes was detected in all isolates in various combinations with *oqx*A and *oqx*B present. Other quinolone resistance genes detected in *Klebsiella pneumoniae* isolates were *qnr*B2, *aac(*6′*)Ib-cr* and *qnr*S1. Apart from *Klebsiella pneumoniae* ST348, all other *Klebsiella pneumoniae* isolates harboured aminoglycoside resistance genes. The *fos*A gene, conferring resistance to fosfomycin, was detected in all *Klebsiella pneumoniae* isolates.

**Table 3:** Sequence type, beta-lactamases genes, plasmid replicon type and pMLST of 38 ESBL-producing *Klebsiella pneumoniae* isolates from neonates.

SNO	SPECIMEN	ST	Beta-lactamases	IncTy	pMLST
1	BLOOD	ST101	*bla*_CTX-M-15_*, bla*_SHV-1_*, bla*_TEM-1B_	IncFIA, IncFIB, IncR	F-:A16:B-
2	BLOOD	ST348	*bla*_CTX-M-15_*, bla*_SHV-11_*, bla*_TEM-1B_	IncFII, IncR	K5:A-:B-
3	BLOOD	ST348	*bla_CTX-M-15_, bla*_SHV-11_	IncFIB, IncR	K5:A-:B-
4	BLOOD	ST35	*bla*_CTX-M-15_*, bla*_SHV-33_*, bla*_TEM-1B_*, bla*_SCO-1_	IncFII, IncFIB, IncR	K12:A-:B-
5	BLOOD	ST35	*bla*_CTX-M-15_*, bla*_SHV-33_*, bla*_TEM-1B_	IncFII, IncFIB, IncR	K12:A-:B-
6	BLOOD	ST35	*bla*_SCO-1_*, bla*_SHV-33_*, bla*_CTX-M-15_*, bla*_TEM-1B_	IncFII, IncFIB, IncR	K12:A-:B-
7	BLOOD	ST45	*bla*_CTX-M-15_*, bla*_SHV-1_	IncHI1B, IncFIB	unknown
8	BLOOD	ST45	*bla*_CTX-M-15_*, bla*_SHV-1_	IncHI1B, IncFIB	unknown
9	BLOOD	ST45	*bla*_CTX-M-15_*, bla*_SHV-1_*, bla*_TEM-1B_*, bla*_OXA-1_	IncHI1B, IncFIB	unknown
10	BLOOD	ST45	*bla*_CTX-M-15_*, bla*_SHV-1_	IncHI1B, IncFIB	unknown
11	BLOOD	ST45	*bla*_CTX-M-15_*, bla*_SHV-1_*, bla*_OXA-1_	InFIB, IncHI1B	Unknown
12	BLOOD	ST45	*bla*_CTX-M-15_*, bla*_SHV-1_*, bla*_OXA-1_	IncHI1B, IncFIB	unknown
13	BLOOD	ST45	*bla*_CTX-M-15_*, bla*_SHV-1_*, bla*_TEM-1B_*, bla*_OXA-1_	IncHI1B, IncFIB	unknown
14	BLOOD	ST48	*bla*_CTX-M-15_*, bla*_SHV-1_*, bla*_TEM-1B_*, bla*_OXA-1_	IncFII, IncFIB	K5:A-:B-
15	BLOOD	ST48	*bla*_CTX-M-15_*, bla*_SHV-1_*, bla*_TEM-1B_*, bla*_OXA-1_	IncFII, IncFIB	K5:A-:B-
16	SWAB	ST101	*bla*_CTX-M-15_*, bla*_SHV-1_*, bla*_TEM-1B_	IncFIA, IncFIB, IncR	F-:A16:B-
17	SWAB	ST14	*bla*_CTX-M-15_*, bla*_SHV-28_*, bla*_TEM-1B_	InFII, IncR	K1:A-:B-
18	SWAB	ST17	*bla*_CTX-M-15_*, bla*_SHV-11_*, bla*_OXA-1_	IncHI1B, IncFIB	unknown
19	SWAB	ST20	*bla*_CTX-M-15_*, bla*_SHV-83_*, bla*_OXA-1_	IncHI1B, IncFIB	Unknown
20	SWAB	ST2268	*bla*_CTX-M-15_*, bla*_SHV-1_*, bla*_TEM-1B_,	IncFIB	Unknown ST
21	SWAB	ST348	*bla*_CTX-M-15_*, bla*_SHV-11_*, bla*_TEM-1B_	IncFII, IncR	K5:A-:B-
22	SWAB	ST348	*bla*_CTX-M-15_*, bla*_SHV-11_*, bla*_TEM-1B_	IncFII	K5:A-:B-
23	SWAB	ST35	*bla*_CTX-M-15_*, bla*_SHV-33_*, bla*_TEM-1B_*, bla*_SCO-1_	IncFII, InFIB, IncR	K12:A-:B-
24	SWAB	ST45	*bla*_CTX-M-15_*, bla*_SHV-1_*, bla*_TEM-1B_*, bla*_OXA-1_	InFIB,IncFII, IncFR, IncFIA	K1:A-:B-
25	SWAB	ST45	*bla*_CTX-M-15_*, bla*_SHV-1_*, bla*_TEM-1B_*, bla*_OXA-1_	InFIB,IncFII, IncFR, IncFIA	K10:A10:B-
26	SWAB	ST45	*bla*_CTX-M-15_*, bla*_SHV-1_*, bla*_TEM-1B_*, bla*_OXA-1_	IncFII, IncFIB, IncR	K5:A-:B-
27	SWAB	ST45	*bla*_CTX-M-15_*, bla*_SHV-1_*, bla*_TEM-1B_*, bla*_OXA-1_	IncFII, IncFIB, IncR	K5:A-:B-
28	SWAB	ST45	*bla*_CTX-M-15_*, bla*_SHV-1_	IncHI1B, IncFIB	unknown
29	SWAB	ST45	*bla*_CTX-M-15_*, bla*_SHV-1_*, bla*_OXA-1_	IncFII,IncHI1B, IncFIB, IncR	K5:A-:B-
30	SWAB	ST45	*bla*_CTX-M-15_*, bla*_SHV-1_*, bla*_TEM-1B_*, bla*_OXA-1_	IncHI1B, IncFIB	Unknown
31	SWAB	ST45	*bla*_CTX-M-15_*, bla*_SHV-1_*, bla*_TEM-1B_*, bla*_OXA-1_	IncHI1B, IncFIB	Unknown
32	SWAB	ST45	*bla*_CTX-M-15_*, bla*_TEM-1B_*, bla*_OXA-1_	IncFII,IncHI1B, IncFIB, IncR	K5:A-:B-
33	SWAB	ST45	*bla*_CTX-M-15_*, bla*_SHV-1_*, bla*_TEM-1B_*, bla*_OXA-1_	InFIB,IncFII, IncFR, IncHI1B	K1:A-:B-
34	SWAB	**ST45**	*bla*_CTX-M-15_*, bla*_SHV-1_*, bla*_TEM-1B_*, bla*_OXA-1_	IncFII, IncFIB, IncR	K5:A-:B-
35	SWAB	ST711	*bla*_CTX-M-15_*, bla*_SHV-83_*, bla*_TEM-1B_*, bla*_OXA-1_	No inc type detected	
36	SWAB	ST873	*bla*_CTX-M-15_*, bla*_SHV-27_*, bla*_TEM-1B_	IncFII, IncFIB	K1:A-:B-
37	SWAB	**Unknown**	*bla*_CTX-M-15_*, bla*_SHV-1_*, bla*_TEM-1B_*, bla*_OXA-1_	InFIB,IncFII, IncFR, IncHI1A	K5:A-:B-
38	SWAB	**Unknown**	*bla*_CTX-M-15_*, bla*_TEM-1B_*, bla*_OXA-1_	?	

ST: sequence type, Inc; Incompatibility, pMLST; plasmid multi locus sequence type.

For the 10 *Escherichia coli* genomes sequenced, ST648 (n = 5) was commonly detected. Other STs detected included ST131 (n = 2), ST 617(n = 1), ST405 (n = 1) and an *Escherichia coli* isolate with a novel ST designation. All *E.coli* strains harboured *bla*_CTX-M-15_ and *aac(*6′*)Ib-cr* genes except for the ST617 isolate which only harboured a *bla*_CTX-M-15_ resistance allele.

An *Enterobacter cloacae* from a swab was typed as ST93, while another isolate obtained from blood was classified as ST116. Both *Enterobacter cloacae* isolates harboured IncHI2 plasmids were assigned to the plasmid (p) MLST ST1. Resistance genes detected in *Enterobacter cloacae* included *bla*_ACT-7_, *bla*_TEM-1B_, *bla*_OXA-1_ and *bla*_CTX-M-15_. The ST93 strain did not harbor a *qnr*B1 gene but carried *sul2*, *dfrA1* and *aadA2* genes that were not present in ST116. The three *Acinetobacter baumannii* isolates were members of ST1470 and ST405 (n = 2) and carried carbapenemase resistance genes *bla*_NDM-1,_ *bla*_OXA-58_, *bla*_OXA-69_, *bla_CARB-8_* and *bla_OXA-69_* (Table 4). IncF plasmids of pMLST K5 and FI were predominant in *Klebsiella pneumoniae* strains and *Escherichia coli* respectively, while for *Enterobacter* spp. the predominant plasmid was IncHI2.

**Table 4:** Sequence type, beta-lactamases genes, plasmid replicon type and pMLST of 15 ESBL-producing *isolates* from neonates.

SNO	SPECIMEN	Isolate	ST	Beta-lactamases	Inc type	pMLST
1	SWAB	*A. baumanii*	ST405	*bla_ADC-25_, bla*_OXA-69_	NIL	
2	SWAB	*A. baumanii*	ST1470	*bla*_NDM-1_*, bla*_OXA-58_	NIL	
3	SWAB	*A. baumanii*	ST405	*bla*_OXA-69_*, bla*_CARB-8,_*bla*_ADC-25_	NIL	
4	BLOOD	*E. cloacae*	ST116	*bla*_CTX-M-15_*, bla*_TEM-1B_*, bla*_OXA-1_*, bla*_ACT_	IncHI2A, IncHI2	ST-1
5	SWAB	*E. cloacae*	ST93	*bla*_CTX-M-15_*, bla*_TEM-1B_*, bla*_OXA-1_*, bla*_ACT-7_	IncHI2A, IncHI2	ST-1
6	SWAB	*E. coli*	ST131	*bla*_CTX-M-15_	IncFIA, IncFIB, IncFII	F1:A1:B16
7	SWAB	*E. coli*	ST131	*bla*_CTX-M-15_*bla*_OXA-1_	IncFIA, IncFIB, IncFII	F1:A1:B16
8	SWAB	*E. coli*	ST405	*bla*_CTX-M-15_*, bla*_TEM-1B_*, bla*_OXA-1_	IncFII, IncFIA, IncFIB	F1:A1:B16
9	SWAB	*E. coli*	ST617	*bla*_CTX-M-15_	IncFII, IncFIB, IncFIA	F1:A2:B33
10	SWAB	*E.coli*	ST648	*bla*_CTX-M-15_*, bla*_TEM-1B_*, bla*_OXA-1_	IncI2,IncFIA, IncFIB, IncFII	F1:A6:B20
11	SWAB	*E. coli*	ST648	*bla*_CTX-M-15_*, bla*_TEM-1B_*, bla*_OXA-1_	IncFII, IncI1, IncFIA	F1:A16:B-
12	SWAB	*E. coli*	ST648	*bla*_CTX-M-15_*bla*_TEM-1B_*, bla*_OXA-1_	IncI2, Col156, IncFIA, IncFIB, IncFII	F1:A6:B20
13	SWAB	*E. coli*	ST648	*bla*_CTX-M-15_*bla*_TEM-1B_*, bla*_OXA-1_	IncQ1, IncFIA, IncY, IncFIB	F-:A1:B32
14	SWAB	*E. coli*	ST648	*bla*_CTX-M-15_*bla*_TEM-1B_*, bla*_OXA-1_	IncQ1, IncFIA, IncY, IncFIB	F-:A1:B32
15	SWAB	*E. coli*	Unknown	*bla*_CTX-M-15_*bla*_TEM-1B_*, bla*_OXA-1_	IncI1, IncQ, IncFIA, IncFIB, Col(BS512)	F1:A1:B49, IncI1=ST 49

*E. coli*; *Escherichia coli*, *E. cloacae; Enterobacter cloacae*, *A. baumanii*; *Acinetobacter baumanii,* ST: sequence type, Inc; Incompatibility, pMLST; plasmid multi locus sequence type.

### 3.4 Virulence genes of *Klebsiella pneumoniae*

**Fig. 1:**
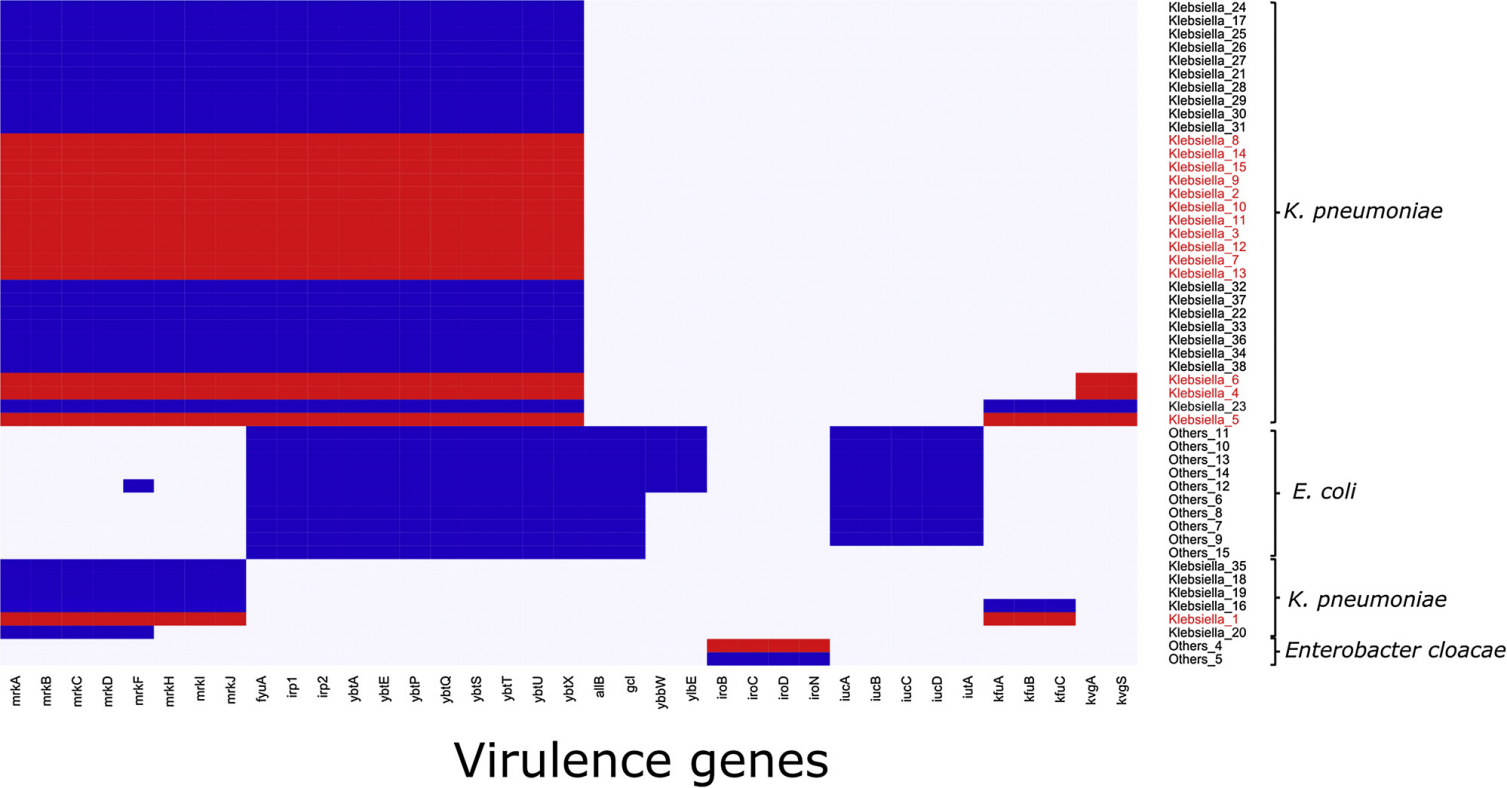
Predicted virulence factors of sequenced ESBL-PE *Enterobacteriaceae*. Legend: Blue, gene present; grey, gene absent (For interpretation of the references to colour in this figure legend, the reader is referred to the web version of this article).

*Klebsiella pneumoniae* isolates harboured up to four different virulence-associated determinants (Fig. 1). The yersiniabactin operon (*ybtAEPQSTUX, irp1, irp2, fyuA*) a metallobactin involved in iron acquisition was detected in 32/38 (84.2%) of the examined *Klebsiella pneumoniae* isolates. Other isolates harbouring yet another operon involved in iron acquisition (*kfuABC*), which was detected only in 4/38 isolates. Genes associated with pilus formation (*mrkABCDFHIJ*) were present in all but one of the (42i) *Klebsiella pneumoniae* examined. Four isolates harboured the *kvgAS* two-component system involved in sensing iron-limiting conditions and free radical stress.

All *Klebsiella pneumoniae* isolates of sequence types ST14, ST348, ST35, ST45, ST48 and ST873 (n = 30) harboured a combination of yersiniabactin and the pili genes (*mrkABCDFHIJ*), regardless of whether they had been isolated from blood or umbilical rectal swabs (Supplementary Table S1). The aerobactin genes (*iucABCD, iutA*), also required for siderophore-based iron uptake, were detected only in *Escherichia coli* (Fig. 1).

### 3.5 Transmission of *Klebsiella pneumoniae*. Isolates to the blood

Twelve pairs of ESBL-PE between neonates blood and neonate swab were detected. In eight neonates, blood culture and neonatal swab isolates depicted identical sequence types. Single nucleotide polymorphisms were identified between these pairs to detect close relationship that indicates a transmission to the blood. Four pairs (50%) depicted very low SNP counts (<20 SNPs) indicating the isolates are of clonal origin. In the other pairs, higher SNP counts were identified, indicating some relationship (Table 5).

**Table 5:** SNP analysis of the blood/swab pairs.

ID NO	BLOOD	ST	Replicon type	ID NO	SWAB	ST	Replicon type	SNP count to blood culture isolate
**5301**	*Kleb.pneu*	45	IncHI1B, IncFIB	140/016.ii	*E.coli*	131	IncFIA, IncFIB, IncFII	Other species, not performed
**6321**	*Kleb.pneu*	48	IncFII, IncFIB	6273	*Kleb.pneu*	48	NA	13
**6331**	*Kleb.pneu*	101	IncFIA, IncFIB, IncR	149/016.11	*Kleb.pneu*	101	IncFIA, IncFIB, IncR	127
**6353**	*Kleb.pneu*	35	IncFII, IncFIB, IncR	036/016	*Kleb.pneu*	35	IncFII, InFIB, IncR	17
**6854**	*Kleb.pneu*	45	IncHI1B, IncFIB	153/016	*Kleb.pneu*	**101**	InFIB, IncFII, IncFR, IncHI1A	Other sequence type, not performed
**6875**	*Kleb.pneu*	348	IncFII, IncR	47	*Kleb.pneu*	348	IncFII, IncR	34
**6881**	*Kleb.pneu*	45	IncHI1B, IncFIB	154/016	*Kleb.pneu*	348	IncFII	Other sequence type, not performed
**6895**	*Kleb.pneu*	45	InFIB, IncHI1B	66	*Kleb.pneu*	45	IncFII,IncHI1B, IncFIB, IncR	72
**7421**	*Kleb.pneu*	45	IncHI1B, IncFIB	31	*Kleb.pneu*	45	IncHI1B, IncFIB	75
**8824**	*Kleb.pneu*	45	IncHI1B, IncFIB	58	*Kleb.pneu*	45	IncHI1B, IncFIB	6
**8900**	*Kleb.pneu*	45	IncHI1B, IncFIB	92	*Kleb.pneu*	45	IncHI1B, IncFIB	4

Kle. pneu: *Klebsiella pneumoniae.*

### 3.6 Predictors of ESBL-PE neonatal sepsis

We investigated different factors to establish whether they could predict ESBL-PE neonatal sepsis. The median age of neonates with ESBL-PE neonatal sepsis was 6 days (IQR: 4–8) while that of ESBL-negative neonatal sepsis was 5 days (IQR: 5–10 days), p = 0.139. It was observed that of 39 neonates admitted at NICU, nine (23.1%) had ESBL-PE neonatal sepsis compared to 23 (8.7%) of 269 neonates admitted at premature unit (p = 0.009). Furthermore, it was observed that of 166 neonates colonized by ESBL-PE, 26 (15.7%) were found to be infected by ESBL-PE as compared to only six (4.35%) neonates with no ESBL-PE colonization (p = 0.003). Using multivariable logistic regression analysis, factors that predicted neonatal ESBL-PE sepsis included: admission at NICU (p = 0.021), positive ESBL-PE colonization of the mother/guardian (p = 0.009) and positive neonate ESBL-PE colonization (p = 0.021) (Table 6).

**Table 6:** Factors associated with ESBL-PE neonatal sepsis among 304 neonates with clinical sepsis at BMC.

Variable	ESBL Positive	ESBL Negative	OR [95% CI]	p-value	OR [95% CI]	p-value
	Median/ n (%)	Median/ n (%)				
***Age in days***	6 IQR (4-8)	5 IQR (3-10)	0.94 [0.88 - 1.02]	0.139	0.93 [0.87-1.02]	0.117
***Sex***						
Female	17 (8.9)	175 (91.15)	1			
Male	15 (13.4)	97 (86.6)	1.59 [0.76 - 3.33]	0.217	1.5 [0.69-3.4]	0.286
***Gestation age***	38 IQR (36-40)	39 IQR (37-40)	1.33 [0.58-3.01]	0.501		
***Body weight***	2.7 IQR (2.3-3)	2.9 IQR (2.1-3.3)	0.79 [0.48-1.29]	0.348		
**Admission**						
BMC	15 (12.9)	101 (87.1)	1			
Referral	17 (9.04)	171 (90.9)	0.7 [0.3-1.4]	0.286		
***Poor feeding***						
No	19 (11.6)	145 (88.4)	1			
Yes	13 (9.3)	127 (90.7)	1.28 [0.61 - 2.69]	0.516		
***Convulsions***						
No	27 (10.3)	236(89.7)	1			
Yes	5 (12.2)	36 (87.8)	1.21 [0.44 - 3.36]	0.709		
***Skin pustule***						
No	27 (10.5)	230 (89.5)	1			
Yes	5 (10.6)	42(89.4)	1.01 [0.36 - 2.78]	0.978		
***Lethargy***						
No	19 (9.6)	178 (90.3)	1			
Yes	13 (12.2)	94 (87.9)	1.3 [0.61 – 2.74]	0.498		
**Antibiotic use**						
No	18 (8.11)	204 (91.9)	1			
Yes	14 (17.1)	68 (82.9)	2.33 [1.1-4.94]	0.027	1.84 [0.82-4.12]	0.139
**Admission**						
PREM	23 (8.7)	242 (91.3)	1			
NICU	9 (23.1)	30 (79.9	3.16 [1.34-7.45]	0.009	3.02 [1.19-7.69]	0.021
**Swab ESBL**						
No	6 (4.35)	132 (95.7)	1			
Yes	26 (15.7)	140 (84.3)	4.09 [1.62-10.24]	0.003	3.08 [1.19-7.99]	0.021
**Stool ESBL**						
No	15 (6.9)	203 (93.1)	1			
Yes	17 (19.8)	69 (80.23)	3.33 [1.58-7.03]	0.002	2.87 [1.3-6.33]	0.009
**Maternal fever**						
No	10 (9.5)	95 (90.5)	1			
Yes	22 (11.1)	177 (88.9)	1.18 [0.53-2.59]	0.679		
**PROM**						
No	22 (10.6)	186 (89.4)	1			
Yes	10 (10.4)	86 (89.6)	0.98 [0.44-2.17]	0.966		
**Maternal antibiotics**						
No	16 (10.1)	143 (89.9)	1			
Yes	16 (11)	129 (88.9)	1.11 [0.53-2.31]	0.783		

### 3.7 Predictors of ESBL-PE neonatal colonization

A total of 166 (54.6%; 95%CI: 49–60.1) neonates were colonized by ESBL-PE. Following univariate analysis, out of the 82 neonates with history of antibiotic use prior being admitted at PREM/NICU, 54(65.9%) were found to be colonized by ESBL-PE as compared to 112 (50.5%) of those without history of antibiotic use (P = 0.017). It was further noted that neonates born from mothers who were colonized by ESBL-PE were more likely to be colonized than those born from mothers with no ESBL-PE colonization (68.6% vs. 49.1%, OR: 2.27, 95%CI 1.34–3.84, P = 0.002). Using multivariable logistic regression analysis, predictors of neonatal ESBL colonization were history of antibiotic use (P = 0.048) and positive ESBL-PE colonization of the mother (P = 0.005) (Table 7).

**Table 7:** Factors associated with neonatal ESBL-PE colonization among 304 neonates with clinical sepsis at BMC.

Variable	ESBL Positive	ESBL Negative	OR [95% CI]	p-value	OR [95% CI]	p-value
	Median/ n (%)	Median/ n (%)				
***Age in days***	6 IQR (3-10)	5 IQR (3-9)	1.01 [0.98 - 1.05]	0.529		
***Sex***						
Female	60 (53.6)	52 (46.4)	1			
Male	106 (55.2)	86 (44.8)	0.94 [0.59 - 1.49]	0.782		
**Admission**						
BMC	60 (51.7)	56 (48.3)	1			
Referral	106 (56.4)	82 (43.6)	1.2 [0.8-1.9]	0.428		
***Birth weight (kg)***	2.9 IQR (2.3-3.3)	2.8 IQR (2.1-3.2)	1.07 [0.79-1.44]	0.654		
***Body temperature***	37.85 IQR (37-38)	37.85 IQR (37-38.3)	0.96 [0.81-1.15]	0.662		
**Oxygen Sat (%)**	93 IQR (91-96)	93 IQR (88-96)	1.02 [0.99-1.05]	0.211	1.03 [0.99-1.06]	**0.113**
***Skin pustule***						
No	140 (54.5)	117 (45.5)	1			
Yes	26 (55.3)	21 (44.7)	1.03 [0.55 - 1.93]	0.915		
**Umbilical discharge**						
No	132 (54.3)	111 (45.7)	1			
Yes	34 (55.7)	27 (44.3)	1.06 [0.61-1.86]	0.843		
**History of antibiotic-baby**						
No	112 (50.5)	110 (49.6)	1			
Yes	54 (65.9)	28 (34.2)	1.89 [1.12-3.21]	0.017	1.73 [1-2.97]	**0.048**
**Admission**						
PREM	141 (53.2)	124 (46.8)	1			
NICU	25 (64.1)	14 (35.9)	1.57 [0.78-3.15]	0.205	1.89 [0.9-3.95]	**0.092**
**Maternal fever**						
No	56 (53.3)	49 (46.7)	1			
Yes	110 (55.3)	89 (44.7)	1.08 [0.67-1.73]	0.746		
**PROM**						
No	116 (56)	91 (43.9)	1			
Yes	50 (51.6)	47 (48.5)	0.83 [0.51-1.35]	0.464		
**Maternal antibiotics**						
No	82 (51.6)	77 (48.4)	1			
Yes	84 (57.9)	61 (42.1)	1.29 [0.82-2.03]	0.266	1.08 [0.67-1.75]	**0.739**
**Stool ESBL**						
No	107 (49.1)	111 (50.9)				
Yes	59 (68.6)	27 (31.4)	2.27 [1.34-3.84]	0.002	2.19 [1.26-3.79]	**0.005**

### 3.8 Predictors of the outcome

Out of 304 neonates, 55 (18.1%, 95% CI 13.4–22.4) died. The median oxygen saturation of neonates who died was 92% (IQR 82–95), while that of neonates who were discharged alive was 93%, IQR: 91–96, (P = 0.003). The mortality rate was significantly higher in neonates infected by ESBL-PE than those infected by other bacteria and/or negative cultures (34.4% vs. 16.2%, p = 0.014). On multivariable logistic regression analysis, predictors of death included being referred from other centres (p = 0.004), being admitted at NICU (p = 0.015) and positive ESBL-PE neonatal sepsis (p = 0.011) Table 8.

**Table 8:** Factors associated with outcome among 304 neonates admitted with clinical sepsis at BMC.

Variable	Death	Discharge Alive	OR [95% CI]	p-value	OR [95% CI]	p-value
	Median/ n (%)	Median/ n (%)				
***Age in days***	7 IQR (4-10)	5 IQR (3-9)	1.03 [0.98 - 1.07]	0.229	1.04[0.99-1.09]	0.103
***Gestation age(weeks)***	38 IQR (35-40)	39 IQR (37-40)	0.92[0.85-0.99]	0.040	0.95 [0.87-1.0]	0.312
**Oxygen Sat (%)**	92 IQR (82-95)	93 IQR (91-96)	0.95[0.92-0.98]	0.003	1.9 [0.9-3.7]	0.083
***Body weight***	2.8 IQR (2-3.2)	2.9 IQR (2.3-3.2)	0.78[0.52-1.14]	0.188		
***Admission***						
*BMC*	12(10.3)	104(89.7)	1			
*Referral*	43(22.9)	145(77.1)	2.6[1.3-5.1]	0.007	2.9 [1.4-6.1]	0.004
***Poor feeding***						
No	31 (18.9)	133 (81.1)	1			
Yes	24 (17.1)	116 (82.9)	1.13[0.62 - 2.03]	0.691		
***Convulsions***						
No	52 (19.8)	211(80.2)	1			
Yes	3 (7.3)	38 (92.7)	0.32 [0.09 – 1.08]	0.066	0.3[0.1-1.1]	0.063
***Lethargy***						
No	33 (16.8)	164 (83.3)	1			
Yes	22 (20.6)	85(79.4)	1.29 [0.71 – 2.34]	0.411		
***Jaundice***						
No	39 (17.7)	181 (82.3)	1			
Yes	16 (19.1)	68 (80.9)	1.09 [0.57 - 2.08]	0.789		
**Ward**						
PREM	40(15.1)	225(84.9)	1			
NICU	15(38.5)	24(61.5)	3.5[1.69-7.28]	0.001	2.7[1.2-6.0]	0.015
**Blood culture**						
Negative	34(15.1)	191(84.9)	1			
Positive	21(26.6)	58(73.4)	2.03[1.09-3.77]	0.024		
**Bacteria/Gram reaction**						
Negative culture	37(15.2)	207(84.8)	1			
Gram positive	5(35.7)	9(64.3)	3.12[0.98-9.79]	0.053		
Gram negative	13(28.3)	33(71.7)	2.2 [1.06-4.58]	0.034		
**ESBL-PE Sepsis**						
No	44(16.2)	228(83.8)	1			
Yes	11(34.4)	21(65.63)	2.71[1.22-6.03]	0.014	3.2[1.3-7.7]	0.011
**Swab ESBL**						
No	21(15.2)	117(84.8)	1			
Yes	34(20.5)	132(79.5)	1.44[0.79-2.61]	0.237		

NB: Blood culture and Bacteria/gram reaction were not subjected on multivariable analysis due to collinearity with ESBL-PE sepsis.

On survival analysis, neonates infected with ESBL-PE (HR: 2.2, 95%CI: 1.13–4.26, P = 0.019) and neonates admitted at NICU (HR: 3.07, 95%CI 1.69–5.56, P < 0.001) had significantly higher mortality rates than their counterpart groups. By multivariate cox regression analysis, factors found to predict high rates of mortality were: neonates infected with ESBL-PE (HR:2.4, 95%CI:1.2–4.8, p = 0.014), neonates admitted at NICU (HR:2.2, 95%CI:1.2.2–4.4, P = 0.018), increase in age (HR:1.05, 95%CI:1.01–1.1, P = 0.021) and those referred from other centres (HR:2.4, 95%CI:1.3–4.6, p = 0.008).

## 4 Discussion

The current study has demonstrated that ESBL-PE contributed to half of the blood culture isolates obtained from neonates in a tertiary hospital in Mwanza, Tanzania. The overall prevalence of the laboratory confirmed sepsis (20%) in the present study is lower than the 50% reported previously from the same centre (Kayange et al., 2010). The difference is probably the result of prophylactic use of broad-spectrum antibiotics in the current cohort. In addition, the proven occurrence of sepsis is also significantly lower than that observed in Mulago National hospital, Kampala Uganda (Mugalu et al., 2006). However, the level of sepsis in the current study is comparable to that observed in Muhimbili national hospital neonatal unit (Mhada et al., 2012).

In low-income countries the major burden of sepsis is borne by children, who are 18 times more likely to die before the age of five than those children in higher-income countries, with infections being a significant source of this disparity (Chan and Lake, 2012). We found that admission to NICU, colonization of neonates, and their mothers/guardians with ESBL-PE were independent predictors of ESBL-PE neonatal sepsis. Neonates infected with ESBL-PE had significantly higher mortality and the majority had severe illness requiring NICU admission. The practice in the neonatal unit/NICU considers carbapenem treatment as the last resort antibiotic. The sad fact of substantial mortality in neonates suffering from blood stream infection due to bacteria with ESBL enzymes that has been demonstrated in the study may very well be a consequence of the unavailability of antimicrobial agents active against those bacteria or due to delay initiation of the appropriate therapy. The slow turnaround times of current blood culture tests lead to a considerable delay in initiating of the appropriate antibiotic treatment and thereby promotes mortality among neonates infected with ESBL-PE. In the current study, the preliminary blood culture results were only obtained after 48 h, with final results for the positive culture given only after 72 h, with incubation for 7 days to confirm negative culture in the majority of cases. The introduction of the semi-automated assays will improve turnaround time of the blood culture and significantly save lives of neonates infected with ESBL-PE. Clinicians should initiate appropriate treatment to high-risk neonates especially those admitted in NICU.

Despite being a cross-sectional study, the study has highlighted important factors that might predict ESBL-PE neonatal sepsis. Previous studies (Kritsotakis et al., 2011; Le et al., 2008; Mshana et al., 2016; Sandiumenge et al., 2006) have demonstrated that exposure to antibiotics can lead to infections/colonization with ESBL-PE, this was also the case in the present study.

As observed in previous studies (Chacha et al., 2014; Kayange et al., 2010), *Klebsiella pneumoniae* was the predominant species isolated. However, unlike a previous study where ST14 was predominant (Mshana et al., 2013), in the current study ST45 has been found to be the most common clone. We conclude that the majority of ESBL-PE sepsis are endogenous since isolates of *Klebsiella pneumoniae* ST45 was both a predominant colonizer of the neonate microflora as well as causing infections at the same time. These data show that most ESBL-PE neonatal sepsis within a given time period might be caused by clonally related isolates as observed in the current study as evidenced by the fact that 50% of paired isolates (blood: neonatal swab) with similar sequence types were clonal. It should be noted that, these isolates could also originate from guardians/mothers, environment or contaminated hands of healthcare workers, underscoring a follow-up exploration study for other sources of neonates colonization. It should be emphasized that hand hygiene can reduce healthcare-associated infections, including those caused by ESBL-PE (Allegranzi and Pittet, 2009). Interestingly, ST45 isolates either colonizing the neonates or associated with neonate infections, harboured different plasmid replicon types and indicate that these strains might have either evolved separately or promiscuously acquired plasmids from different sources. Thus, blood isolates harboured IncFIB and IncHI1 plasmids of hitherto unclassified pMLST types, while ST45 strains colonizing the neonates had IncFII, IncFIB and IncR plasmids.

The majority of *Klebsiella pneumoniae* isolates harboured genes for siderophores involved in sequestering iron from the environment. The presence of siderophores have been linked to community infections (Holt et al., 2015) emphasizing the broad occurrence of ESBL-PE in our setting. Surprisingly only two *Escherichia coli* strains, both of which were not ESBL, were isolated from the blood despite the fact that about 70% and 36% of guardians/mothers and neonates were colonized by ESBL-producing *Escherichia coli.* The intrinsic ability of *Klebsiella pneumoniae* to survive in the environment and its ability to survive in the serum, through siderophores-dependent iron acquisition, could partially account for its transmission to neonates and development of blood stream infections (Podschun and Ullmann, 1998). Indeed in the majority of the isolates sequenced, virulence factors predisposing to infections were detected.

As in previous studies (Bhutta et al., 2010; Mshana et al., 2011, 2016; Seni et al., 2016) in the hospital and community among humans and animals, 90% of isolates were found to carry *bla*_CTX-M-15_. *Acinetobacter baumannii* carrying a *bla*_NDM-1_ gene was detected as a colonizing isolate. This is worrisome because the inappropriate use of meropenem could promote selective growth and hence increase challenges in the management of the neonatal sepsis in low-income countries. Furthermore the *bla*_NDM-1_ might translocate to conjugative plasmids and be transmitted to other Enterobacteriaceae through horizontal genetic transfer (Struelens et al., 2010).

The limitations of this study include the following: Because of the low number of culture-positive blood samples we decided to compare those with ESBL-PE neonatal sepsis and other neonates assuming that all neonates were, from the onset, equally at risk of getting ESBL-PE neonatal sepsis. Being a single center, the results may not be generalizable. A prospective multicenter cohort study would have been more suitable. Another limitation was the lack of colonization and mortality data from neonates without signs and symptoms of neonatal sepsis for comparative analysis. Finally, not all isolates underwent molecular characterization including those colonizing the mother/guardians. Future studies should consider whole genome sequencing of larger numbers of isolates from neonates, guardian/mothers and health care workers to reveal dynamics of transmission and other source of ESBL-PE.

## 5 Conclusion and recommendation

The majority of septic neonates has been shown to be colonized by ESBL producers and among those colonized with strains secreting ESBL enzymes; the majority was shown to be colonized by ESBL-producing strains of *K. pneumoniae*. ESBL-producing *Klebsiella pneumoniae* was the most frequent isolated from blood of neonates at BMC neonatal unit. The virulent isolates of the *Klebsiella pneumoniae* ST45 carrying *bla*_CTX-M-15_ were predominantly infecting and colonizing neonates. Maternal and neonatal ESBL-PE colonization are important factors predicting ESBL neonatal sepsis. Our study demonstrates that neonatal mortality is significantly high in neonates infected with ESBL-PE. There is a need to improve and emphasize on infection control interventions and antimicrobial stewardship because these are cheap inexpensive strategies to control the increase of multi-resistant gram-negative bacteria. Furthermore, rapid diagnostic tests to detect ESBL-PE from neonates’ blood are highly needed in the low-income countries in order to reduce the mortality with ESBL-PE neonatal sepsis. In addition, there is a need to improve turnaround time of blood culture assays for the neonatal units in order to guide antibiotic treatment.

## Competing interests

The authors declare no competing interests.
